# Estimated prevalence of gaming disorder based on ICD-11 and DSM-5 frameworks in a large and representative sample of young Swiss men

**DOI:** 10.1016/j.abrep.2026.100701

**Published:** 2026-04-29

**Authors:** Julia de Ternay, Olivier Simon, Stéphanie Baggio, Joël Billieux

**Affiliations:** a Service Universitaire d’Addictologie de Lyon (SUAL), Hôpital Edouard Herriot, Hospices Civils de Lyon, Lyon, France; b Research on Healthcare Performance (RESHAPE), INSERM U1290, Hospices Civils de Lyon, Lyon, France; c Center for Excessive Gambling, Addiction Medicine, Lausanne University Hospital, Lausanne, Switzerland; d Institute of Psychology, University of Lausanne, Lausanne, Switzerland; e Institute of Primary Health Care (BIHAM), University of Bern, Bern, Switzerland

**Keywords:** Gaming disorder, Gaming addiction, ICD-11, DSM-5, Young adults

## Abstract

*Background*: The International Classification of Diseases, 11th revision (ICD-11), and the Diagnostic and Statistical Manual of Mental Disorders, 5th edition (DSM-5), provide distinct frameworks to assess gaming disorder (GD). We aimed to compare GD prevalence estimates using both frameworks in a representative sample of Swiss males. A secondary objective was to examine the associations between GD, and depressive and anxiety symptoms, across both frameworks.

*Methods:* This was a cross-sectional single-center study at a Swiss military recruitment center. Participants completed a survey assessing DSM-5 and ICD-11 GD criteria. GD was defined as meeting at least five DSM-5 or all three ICD-11 criteria. Depressive and anxiety symptoms were assessed with the two-item Patient Health Questionnaire and the two-item General Anxiety Disorder scale. Weekly gaming time was self-reported. Multivariable logistic regressions explored associations between GD and psychological correlates.

Results: Among the 2,620 participants aged 19.6 ± 1.8 years old, the estimated GD prevalence was 3.83% (95% confidence interval [CI]: 3.13; 4.69) with DSM-5 criteria and 2.48% (95% CI: 1.92; 3.18) with ICD-11 criteria (p < 0.001). In the subsample of past-year gamers (n = 2,164), screening positive for major depressive disorder was associated with GD under both classifications. Screening positive for generalized anxiety disorder was associated with GD only under DSM-5 criteria.

*Conclusion:* These findings support the assumption that the ICD-11 classification provides a more conservative estimated prevalence than the DSM-5. This study also highlights the differences between DSM-5 and ICD-11 frameworks in their associations with mental health correlates.

## Highlights

• Gaming disorder prevalence is higher with DSM-5 than with ICD-11 criteria
• Depression is associated with gaming disorder under both frameworks
• Anxiety is linked to gaming disorder only when using DSM-5 criteria
• ICD-11 may identify fewer but more severe gaming disorder cases

## 1 Introduction

After years of debate, gaming disorder (GD) was officially recognized as an addictive disorder and included in the International Classification of Diseases, 11th revision (ICD-11; World Health Organization, 2024). In contrast, the other widely used classification system for mental health disorders, i.e., the Diagnostic and Statistical Manual of Mental Disorders, 5th edition, text revision (DSM-5-TR; American Psychiatric Association, 2023), has not yet officially acknowledged GD as a formal diagnosis. Instead, GD remains considered a candidate disorder for future versions of the DSM under the label “internet gaming disorder” in its section on emerging conditions. Although criteria to establish a diagnosis differ between the ICD-11 and the DSM-5-TR (Table 1), both frameworks are used to diagnose GD and estimate its prevalence (Fam, 2018, Gao et al., 2022, Kim et al., 2022).

**Table 1:** Comparison of gaming disorder criteria between the International Classification of Diseases, 11th revision (ICD-11), and the Diagnostic and Statistical Manual of Mental Disorders, 5th edition, text revision (DSM-5-TR).

**Gaming Disorder (ICD-11)**	**Internet Gaming Disorder (DSM-5-TR)**	
1. Impaired control over gaming	1. Preoccupation with gaming	6. Continuing to game despite problems
2. Increasing priority given to gaming over other activities	2. Withdrawal symptoms when gaming is taken away or not possible	7. Deceiving family members or others about the amount of time spent on gaming
3. Continuation of gaming despite negative consequences	3. Tolerance, the need to spend more time gaming to satisfy the urge	8. Use of gaming to relieve negative moods
	4. Inability to reduce playing, unsuccessful attempts to quit gaming	9. Risk, having jeopardized or lost a job or relationship due to gaming
**Presence of all three criteria over a 12-month period**	5. Loss of interest in previously enjoyed activities due to gaming	
**Clinically significant distress/impairment in important areas of functioning**	**At least five of nine criteria over a 12-month period**	

Table 1.

Substantial variability in GD prevalence estimates has been reported across studies, ranging from 0.3% to 17.7% (Kim et al., 2022). This heterogeneity likely reflects the diversity of screening questionnaires used in research, combined with the absence of a reliable tool for clinical diagnosis. Such variability has raised concerns about the potential over-pathologizing of normal gaming behavior, both in epidemiological studies and clinical settings, a key point of contention in the ongoing debate about the formal recognition of GD as a clinical diagnosis (Aarseth et al., 2017, Király and Demetrovics, n.d., Pontes and Griffiths, 2026, van Rooij et al., 2018). Differences in diagnostic criteria may further contribute to this variability. In particular, several DSM-5 criteria such as tolerance-like symptoms, deception, or gaming to escape, have been suggested to inflate prevalence estimates and promote false-positive diagnoses (Castro-Calvo et al., 2021, Ko et al., 2014, Rehbein et al., 2015, Wang and Cheng, 2020). In contrast, criteria such as loss of control, included in both the ICD-11 and DSM-5 frameworks, appear more valid and discriminative (Ino et al., 2025). The delineation of GD as a distinct disorder is further complicated by the frequent co-occurrence of other mental health conditions, particularly depressive disorders (Ostinelli et al., 2021, Yen et al., 2024) and generalized anxiety disorders (Wang et al., 2017, Yen et al., 2024). Symptoms of these conditions may be inadvertently captured by GD criteria in the ICD-11 or DSM-5 classifications, potentially leading to diagnostic overlap or inflation. For instance, in a study based on DSM-5 criteria, 62.3% of individuals who met the threshold of GD also met criteria for depression (Wang et al., 2018). Although both depression and anxiety have been consistently associated with GD, most studies have examined these relationships using a single diagnostic framework, either DSM-5 or ICD-11, rather than testing them across both frameworks. As a result, it remains unclear whether the strength of these associations could differ depending on the classification used.

Given these concerns, an important question is whether one of the two frameworks should be preferred in clinical and research contexts to identify GD. Previous studies comparing DSM-5 and ICD-11 frameworks generally suggest that the ICD-11 applies a higher diagnostic threshold (Higuchi et al., 2021, Nogueira-López et al., 2023, Yen et al., 2022, Yen et al., 2023. However, important methodological limitations remain, since many of these studies have relied on small, convenience-based or clinical samples that may not be representative of the general population, and could lead to biased and unreliable prevalence estimates. Although Nogueira-López et al. (2023) addressed this issue by using a representative sample of Spanish adolescents, their study relied on proxy ICD-11 criteria derived from DSM-5 items, rather than the official ICD-11 definition, which constitutes an important source of measurement bias. Addressing these limitations is essential, as differences between classification systems may reflect not only conceptual distinctions but also methodological artefacts related to measurement and sampling. In a field characterized by substantial heterogeneity in measurement approaches, improving methodological rigor may be as critical as generating novel findings.

In this study, we aimed to provide a robust comparison of DSM-5 and ICD-11 frameworks by using their original criteria in a large and representative sample of young Swiss men, thereby improving the methodological validity of prevalence estimates and associated correlates. From previous studies, we hypothesized that the estimated prevalence of GD would be higher when assessed with DSM-5 criteria than when assessed with ICD-11 criteria. A secondary objective of the study was to test the associations between GD, as defined by DSM-5 and ICD-11 criteria, and depressive and anxiety symptoms. We hypothesized that depressive and anxiety symptoms would be positively associated with GD under both frameworks.

## 2 Methods

### 2.1 Design

This study was a cross-sectional single-center study at a French-speaking military recruitment center in Switzerland.

### 2.2 Sample

The study sample consisted of Swiss French-speaking young men attending mandatory army conscription, which includes the vast majority (98%) of young Swiss men in the relevant age range, between February 2019 and March 2020. All Swiss men undergo this recruitment process without pre-selection, regardless later eligibility for military, civil, or no service. This context allows for broad population coverage and reduces selection bias compared to voluntary samples. Women were not included in the sample because army conscription in Switzerland is not mandatory for them. Participants were eligible for inclusion if they had engaged in at least one online activity in the past year. Exclusion criteria included the inability to provide informed consent or unwillingness to participate in the study.

All conscripts were invited to participate in the framework of workshops on gambling prevention and awareness organized by the Centre for Excessive Gambling at the military recruitment center. After providing written informed consent, participants completed a 10-minute paper-and-pencil survey. Data were collected anonymously to ensure confidentiality. This was an exploratory study using an existing dataset from a previous study, the results of which have already been published (Baggio et al., 2024). All data and materials used are publicly available on the Open Science Framework (OSF): https://osf.io/bvpxf/. Please note that some materials included in this OSF depository (e.g., analytic codes) are associated with the study from Baggio et al (Baggio et al., 2024).

### 2.3 Measures

Participants were first asked whether they had engaged in online gaming, online gambling, social networking, or online sexual activities in the past 12 months (yes/no). They also provided information on their age and level of education (primary, secondary, or tertiary). To evaluate the presence of problematic online gaming, we used the statements that assessed the ICD-11 criteria (World Health Organization, n.d.) and the internet gaming disorder criteria from Section 3 of the DSM-5 (American Psychiatric Association, n.d.) (see Table 1 in the Supplemental Material). Participants were classified as having problematic online gaming if they met all three ICD-11 criteria or at least five DSM-5 criteria. Participants self-reported the amount of time spent gaming during a typical week and a typical weekend. Depressive and anxiety symptoms were assessed with the two-item Patient Health Questionnaire (PHQ-2) (Kroenke et al., 2003) and the two-item General Anxiety Disorder scale (GAD-2) (Kroenke et al., 2007). Participants were considered to have screened positive for major depressive disorder (MDD) if they scored three or higher on the PHQ-2 (Kroenke et al., 2003) and for generalized anxiety disorder (GAD) if they scored three or higher on the GAD-2 (Kroenke et al., 2007).

### 2.4 Statistical analyses

We performed descriptive analyses of the sample and calculated the estimated prevalence of problematic online gaming according to the ICD-11 criteria and the DSM-5 criteria, along with their corresponding 95% confidence intervals (CIs). Categorical variables are presented as counts and percentages, and quantitative variables are expressed as mean ± standard deviation (or median, where appropriate). These estimated prevalence rates were then compared by using a chi-squared test. Standardized effect sizes were calculated with Cohen’s h (Cohen, 1988), which is specifically designed for comparing proportions. According to Cohen’s guidelines, an h value below 0.20 indicates a small effect, values between 0.20 and 0.80 indicate a medium effect, and values above 0.80 indicate a large effect.

In the subsample of participants who reported engaging in gaming at least once in the past year (gamers), we performed two multivariable logistic regression analyses. The dependent variable was whether participants screened positive for problematic online gaming (yes/no), as defined by ICD-11 criteria (Model 1) or DSM-5 criteria (Model 2). Independent variables included positive screening for depressive or anxiety symptoms on the PHQ-2 and GAD-2 (yes/no) and the logarithmic transformation of weekly gaming time.

The multivariable logistic regression analyses were performed only on complete cases for both Model 1 (n = 2,012) and Model 2 (n = 1,953). All statistical analyses were conducted by using R (version 4.1.3, 2022) via RStudio (version 2023–09-1 + 494).

### 2.5 Ethics

All participants were informed about the study and all provided informed consent. The study protocol was approved by the Geneva Cantonal Ethics Committee (no. 2018–02105).

## 3 Results

A total of 2,729 young men were eligible to participate in the study, of whom 109 declined (response rate: 96.0%). The final sample consisted of 2,620 participants (Table 2), with an average age of 19.6 ± 1.8 years. The majority had a secondary level of education (50.7%) and reported having played videogames in the past year (82.6%). On average, participants spent 198.5 ± 269.2 min gaming per week, with a median at 120.0 min. A minority of participants screened positive for MDD based on the PHQ-2 (6.6%) and for a GAD based on the GAD-2 (7.3%). Thirty participants met both DSM-5 and ICD-11 criteria for GD. This result corresponded to 31.6% of those who screened positive according to DSM-5 criteria (n = 95) and 47.6% of those who screened positive according to ICD-11 criteria (n = 63).

**Table 2:** Characteristics of the sample.

		**Gaming in the last 12 months**	
Characteristic	Overall, N = 2,619	No, N = 455	Yes, N = 2,164
**Age**	19.6 (1.8) 19.0	20.1 (2.2) 19.0	19.5 (1.7) 19.0
*Missing*	97	16	81
**Level of education**			
*Primary*	935 (38.1%)	186 (43.5%)	749 (37.0%)
*Secondary*	1,244 (50.7%)	181 (42.3%)	1,063 (52.4%)
*Tertiary*	276 (11.2%)	61 (14.3%)	215 (10.6%)
*Missing*	164	27	137
**Gaming time per week (min)**	198.5 (269.2) 120.0	/	231.7 (276.2) 180.0
*Missing*	49	/	33
**GD according to the DSM-5**	95 (3.8%)	/	95 (4.7%)
*Missing*	141	/	141
**GD according to the ICD-11**	63 (2.5%)	/	63 (3.0%)
*Missing*	75	/	75
**MDD according to the PHQ2**	167 (6.6%)	32 (7.2%)	135 (6.5%)
*Missing*	95	13	82
**GAD according to the GAD2**	183 (7.3%)	31 (7.0%)	152 (7.3%)
*Missing*	101	15	86
Mean (SD) Median; n (%)			

*Note.* Values are shown as n (%) unless otherwise indicated. DSM-5 = the Diagnostic and Statistical Manual of Mental Disorders, 5th edition; ICD-11 = the International Classification of Diseases, 11th revision; MDD = major depressive disorder; PHQ-2 = two-item Patient Health Questionnaire; GAD = generalized anxiety disorder; GAD-2 = two-item General Anxiety Disorder scale. One observation with a missing value to “Gaming in the last 12 months” does not appear in the table.

The estimated prevalence of GD was 3.83% (95% CI: 3.13; 4.69) according to DSM-5 criteria and 2.48% (95% CI: 1.92; 3.18) according to ICD-11 criteria (Table 2). These estimated prevalence rates were significantly different (p < 0.001, Cohen’s h = 0.078) based on a chi-squared test.

In the subsample of participants who reported engaging in gaming at least once in the past year (n = 2164) (Table 2), scoring positive for MDD was a significant risk factor for problematic online gaming according to both the ICD-11 criteria (adjusted odds ratio [aOR]: 4.42, 95% CI: 2.05; 9.11) and the DSM-5 criteria (aOR: 2.37, 95% CI: 1.19; 4.59) (Table 3). Scoring positive for GAD was a risk factor for problematic online gaming based on DSM-5 criteria only (aOR: 3.05, 95% CI: 1.59; 5.68). In addition, problematic gamers reported significantly higher weekly gaming time in both models: aOR: 1.89 (95% CI: 1.46; 2.51) for the ICD-11 criteria and aOR: 2.30 (95% CI: 1.81; 2.96) for the DSM-5 criteria.

**Table 3:** Multivariable logistic regression models used to assess factors associated with a positive gaming disorder screening as defined by ICD-11 criteria (Model 1) or DSM-5 criteria (Model 2) among participants having engaged in gaming in the past year.

	Model 1 (ICD-11)			Model 2 (DSM-5)		
**Characteristic**	**aOR**	**95% CI**	**p-Value**	**aOR**	**95% CI**	**p-Value**
**MDD according to the PHQ-2**						
*Yes*	4.42	(2.05; 9.11)	**<0.001**	2.37	(1.19; 4.59)	**0.012**
**GAD according to the GAD-2**						
*Yes*	1.38	(0.59; 3.03)	0.437	3.05	(1.59; 5.68)	**<0.001**
**Gaming time per week (min)**	1.89	(1.46; 2.51)	**<0.001**	2.30	(1.81; 2.96)	**<0.001**

*Note.* Model 1 (n = 2,012), Model 2 (n = 1,953).

ICD-11 = the International Classification of Diseases, 11th revision; DSM-5-: the Diagnostic and Statistical Manual of Mental Disorders, 5th edition; aOR = adjusted odds ratio; CI = confidence interval; MDD = major depressive disorder; PHQ-2 = two-item Patient Health Questionnaire; GAD = generalized anxiety disorder; GAD-2 = two-item General Anxiety Disorder scale.

## 4 Discussion

The estimated prevalence of problematic online gaming was 3.83% according to the DSM-5 criteria, whereas it was 2.48% according to the ICD-11 criteria. Although the exclusion of a small number of individuals who reported no online activity in the past 12 months (in accordance with our eligibility criteria) may have slightly inflated the estimated prevalence, these figures remain consistent with pooled European GD prevalence reported in recent meta-analyses (Gisbert-Pérez et al., 2026, Stevens et al., 2021). We observed a significantly higher prevalence of GD with the DSM-5 criteria than the ICD-11 criteria, which aligns with previous findings (Borges et al., 2021, Fam, 2018, Higuchi et al., 2021, Nogueira-López et al., 2023, Pontes et al., 2022). However, the magnitude of this difference was relatively small. Although such a difference may be meaningful from a populational-level public health perspective, it remains an empirical question as to whether the choice of diagnostic framework meaningfully affects clinical decision making or treatment eligibility in practice. In treatment seeking individuals with problematic gaming, high concordance between DSM-5 and ICD-11 frameworks has been reported, suggesting that both frameworks similarly identify severe clinical cases (Higuchi et al., 2021). Moreover, differences between DSM-5 and ICD-11 do not appear to constitute a major barrier to diagnosis in specialized clinical settings, where clinicians generally report being able to identify and assess GD (Park et al., 2024). Nevertheless, although DSM-5 and ICD-11 seem to identify overlapping populations, DSM-5 criteria are slightly more easily endorsed. This may reflect concerns that the DSM-5 approach is at risk of conflating clinical cases with highly involved (e.g., several hours a day), yet non-pathological, patterns of gaming (Billieux et al., 2019).

Beyond differences in prevalence estimates, our findings also highlight differences in associations with mental health correlates. Screening positive for MDD was significantly associated with GD under both classifications, whereas GAD was a predictor with DSM-5 criteria only. This finding reinforces evidence of a robust association between depressive symptoms and GD, regardless of the diagnostic framework (Ostinelli et al., 2021, Tateno et al., 2025). Longitudinal studies suggest that depressive symptoms often precede the onset of GD (Guillot et al., 2016, Liu et al., 2021, Teng et al., 2021), although temporal dynamics and potential bidirectional influences remain unclear. Interestingly, the association with MDD may be stronger under the ICD-11 classification. Combined with the lower estimated prevalence using ICD-11 criteria, this may indicate that ICD-11 identifies more severe and functionally impairing cases. In contrast, anxiety symptoms were associated with GD only when we used DSM-5 criteria. At first glance, this might suggest that DSM-5-positive cases were more symptomatic. However, an alternative explanation could be that some DSM-5 criteria are less discriminative, increasing sensitivity at the expense of specificity and potentially inflating prevalence estimates. Supporting this interpretation, criteria such as escapism and preoccupation have been linked to general emotional distress and may also be present in normative gaming behaviors (Kashdan et al., 2014, Ko et al., 2020, Rohde et al., 1990, She et al., 2024). Moreover, over-involvement in gaming may reflect a maladaptive coping strategy in individuals with anxiety (Billieux et al., 2025, Giardina et al., 2024). Taken together, our findings suggest that DSM-5 criteria may be endorsed across a broader spectrum of gaming patterns, ranging from problematic to highly engaged but non-pathological use, whereas ICD-11 criteria may be more likely to capture severe cases with clearer psychopathological features (Billieux et al., 2019, Starcevic et al., 2020). Comparing the profiles of individuals who screen positive for GD under one classification but not the other may help elucidate the respective strengths and limitations of each diagnostic framework.

We acknowledge several limitations of our study. First, the sample consisted exclusively of young Swiss men, which limits the generalizability of our findings to women and to other age or cultural groups. Future studies should replicate this research in women and in more diverse populations. However, the large sample size, its representativeness of young Swiss men, and the high response rate constitute important strengths. Second, we relied on self-report screening tools rather than clinical interviews. Consequently, our findings reflect probable cases rather than formal diagnoses. Third, the use of self-administered questionnaires may have introduced social desirability bias, although the anonymity of the survey likely reduced this risk, as well as recall bias. However, most measures assessed behaviors and symptoms over the past year or more recent periods, which likely limited this risk. Finally, we assessed only anxiety and depressive symptoms and did not examine other psychopathological features. In addition, the instruments used to assess anxiety and depression are short screening tools that may not necessarily have captured the full spectrum of symptoms and features of these disorders. Concerning the study’s cross-sectional design, it appears to be appropriate given our objectives to compare estimated prevalence rates and correlates of GD by using two diagnostic frameworks. Our aim was not to infer causal relationships but to explore associations and diagnostic implications. In this context, the lack of longitudinal data does not undermine the validity of our primary conclusions.

The present study was not conducted to primarily identify novel differences in prevalence or associations, as such patterns have been previously described. Rather, its main contribution lies in strengthening the robustness and interpretability of these findings by addressing key methodological limitations in prior research. In addition, it tested associations between GD and depressive and anxiety symptoms across DSM-5 and ICD-11 frameworks within the same population, an approach that has been scarcely explored. Our findings support the assumption that ICD-11 applies a slightly higher diagnostic threshold than DSM-5, and provide more robust estimates of the associations between each framework and mental health correlates.

## 5 Funding sources

This research did not receive any specific grant from funding agencies in the public, commercial, or not-for-profit sectors.

## CRediT authorship contribution statement

**Julia de Ternay:** Writing – review & editing, Writing – original draft, Visualization, Validation, Project administration, Methodology, Formal analysis, Conceptualization. **Olivier Simon:** Writing – review & editing, Resources, Data curation. **Stéphanie Baggio:** Writing – review & editing, Validation, Resources, Methodology, Data curation, Conceptualization. **Joël Billieux:** Writing – review & editing, Validation, Supervision, Resources, Methodology, Data curation, Conceptualization.

## Declaration of competing interest

The authors declare that they have no known competing financial interests or personal relationships that could have appeared to influence the work reported in this paper.

## Data Availability

Data will be made available on request.

## Appendix A Supplementary data

Supplementary Data 1 Supplemental Material contains a Table of the ICD-11 and DSM-5 criteria assessed by study participants.
