# Supplementary material for: Estimated prevalence of gaming disorder based on ICD-11 and DSM-5 frameworks in a large and representative sample of young Swiss men
Source: Addict Behav Rep. 2026 Apr 29;23:100701. doi: 10.1016/j.abrep.2026.100701 (PMC13145247; doi:10.1016/j.abrep.2026.100701)
Supplement: Supplementary Data 1 — Supplemental Material contains a Table of the ICD-11 and DSM-5 criteria assessed by study participants. [file mmc1.docx]

Supplemental Material

| **Item number** | **French version** |  |  |
| --- | --- | --- | --- |
|  | **Concernant votre utilisation des jeux vidéo sur Internet, au cours des 12 derniers mois...** | **Oui** | **Non** |
| **1** | ... avez-vous été préoccupé par les jeux vidéo (par exemple se remémorer des expériences de jeu passé ou prévoir de jouer, les jeux vidéo devenant l’activité dominante de la vie quotidienne) ? |  |  |
| **2** | ... avez-vous été irritable, anxieux ou triste quand l’accès aux jeux vidéo était supprimé ? |  |  |
| **3** | ... avez-vous tenté de manière infructueuse de contrôler votre participation aux jeux vidéo ? |  |  |
| **4** | ... avez-vous eu besoin de consacrer des périodes de temps croissantes aux jeux vidéo ? |  |  |
| **5** | ... avez-vous joué aux jeux vidéo pour échapper à ou soulager une humeur négative (par exemple des sentiments d’impuissance, de culpabilité ou d’anxiété) ? |  |  |
| **6** | ... avez-vous mis en danger ou perdu une relation affective importante, un emploi ou des possibilités d’étude ou de carrière à cause de votre participation à des jeux vidéo ? |  |  |
| **7** | ... avez-vous menti à votre famille, votre thérapeute ou à d’autres personnes sur l’ampleur de votre utilisation des jeux vidéo ? |  |  |
| **8** | ... avez-vous perdu l’intérêt pour les loisirs et divertissements antérieurs du fait et à l’exception des jeux vidéo ? |  |  |
| **9** | ... avez-vous continué à jouer aux jeux vidéo bien que vous sachiez que cela vous causait des problèmes psychosociaux ? |  |  |
| **10** | ... avez-vous perdu le contrôle sur les jeux vidéo et avez passé plus de temps que prévu à jouer ? |  |  |
| **11** | ... avez-vous donné la priorité aux jeux vidéo de telle manière que les jeux sont devenus l’activité dominante en comparaison de vos autres intérêts dans la vie et de vos activités quotidiennes ? |  |  |
| **12** | ... avez-vous continué à jouer en dépit de conséquences négatives (par exemple, conflits familiaux ou conjugaux, négligence d’amis ou de responsabilités, mauvais résultats scolaires ou professionnels, perte d’emploi, ou problèmes physiques) ? |  |  |
|  | **English version** |  |  |
|  | **Regarding your use of video games on Internet in the past 12 months…** | **Yes** | **No** |
| **1** | ... have you been preoccupied with video games (e.g., thinking about past gaming experiences or anticipating gaming, with gaming becoming the dominant daily activity)? |  |  |
| **2** | ... have you felt irritable, anxious, or sad when access to video games was taken away? |  |  |
| **3** | ... have you made unsuccessful attempts to control your involvement in video games? |  |  |
| **4** | ... have you needed to spend increasing amounts of time playing video games? |  |  |
| **5** | ... have you played video games to escape from or relieve a negative mood (e.g., feelings of helplessness, guilt, or anxiety)? |  |  |
| **6** | ... have you jeopardized or lost a significant relationship, job, or educational or career opportunity because of your gaming? |  |  |
| **7** | ... have you lied to family members, therapists, or others about the extent of your video game use? |  |  |
| **8** | ... have you lost interest in previous hobbies and entertainment activities as a result of, and with the exception of, video games? |  |  |
| **9** | ... have you continued to play video games despite knowing that it caused psychosocial problems? |  |  |
| **10** | ... have you lost control over video gaming and spent more time playing than intended? |  |  |
| **11** | ... have you given increasing priority to video games to the extent that gaming has taken precedence over other life interests and daily activities? |  |  |
| **12** | ... have you continued to play despite negative consequences (e.g., family or partner conflicts, neglecting friends or responsibilities, poor school or work performance, job loss, or physical problems)? |  |  |

Table 1: ICD-11 (in blue) and DSM-5 (in orange) criteria assessed by study participants

ICD-11: International Classification of Disease, 11^th^ edition

DSM-5: Diagnostic and Statistical Manual of Mental Disorders, Fifth Edition
